# Controllable Hysteresis and Threshold Voltage of Single-Walled Carbon Nano-tube Transistors with Ferroelectric Polymer Top-Gate Insulators

**DOI:** 10.1038/srep23090

**Published:** 2016-03-16

**Authors:** Yi-Lin Sun, Dan Xie, Jian-Long Xu, Cheng Zhang, Rui-Xuan Dai, Xian Li, Xiang-Jian Meng, Hong-Wei Zhu

**Affiliations:** 1 Institute of Microelectronics, Tsinghua National Laboratory for Information Science and Technology (TNList), Tsinghua University, Beijing 100084, People’s Republic of China; 2 National Laboratory for Infrared Physics, Shanghai Institute of Technical Physics, Chinese Academy of Sciences, 500Yu Tian Road, Shanghai 200083, China; 3 School of Materials Science and Engineering, State Key Laboratory of New Ceramics and Fine Processing, Key Laboratory of Materials Processing Technology of MOE, Tsinghua University, Beijing 100084, China; 4 Center for Nano and Micro Mechanics, Tsinghua University, Beijing 100084, China

## Abstract

Double-gated field effect transistors have been fabricated using the SWCNT networks as channel layer and the organic ferroelectric P(VDF-TrFE) film spin-coated as top gate insulators. Standard photolithography process has been adopted to achieve the patterning of organic P(VDF-TrFE) films and top-gate electrodes, which is compatible with conventional CMOS process technology. An effective way for modulating the threshold voltage in the channel of P(VDF-TrFE) top-gate transistors under polarization has been reported. The introduction of functional P(VDF-TrFE) gate dielectric also provides us an alternative method to suppress the initial hysteresis of SWCNT networks and obtain a controllable ferroelectric hysteresis behavior. Applied bottom gate voltage has been found to be another effective way to highly control the threshold voltage of the networked SWCNTs based FETs by electrostatic doping effect.

Single-walled carbon nanotubes (SWCNTs) possesses outstanding properties such as high carrier mobility, high ON/OFF ratio and easy solution processability for large-scale production due to their unique one-dimensional structure^1^,^2^,^3^. Meanwhile, SWCNTs have been one of the most promising materials for thin film transistors (TFTs), in which they work as the active channel^4^,^5^. However, the practical utilization of networked SWCNT-FETs is still facing many challenging issues, including the large hysteresis^6^ and the control of the threshold voltage (*V*_*th*_) of SWCNT-FETs^7^,^8^. The large hysteresis found in the transfer characteristics as a function of gate voltage may be attributed to complex interactions between the charges doping into the SWCNTs by the water and oxygen molecules surrounding the surface of the nanotubes^9^,^10^,^11^,^12^ and the field-effect induced charges, which are trapped and located at the surface or bulk of gate transistor dielectrics such as silanol groups on a Si oxide surface^7^,^13^. Recently, many attempts have been devoted to reduce the hysteresis of the SWCNT-FETs such as the passivation of the SWCNTs with PMMA^12^, and the fabrication of SWCNT-FETs by a directed-assembly method^14^. However, these studies are aimed at single-tube transistors, which are involved in a sophisticated device-fabrication process. Moreover, it is almost impossible to reduce the hysteresis of complex networked SWCNTs by the elimination of direct-contacted external charges in networked SWCNT-FETs^15^. Meanwhile, the suboptimal threshold voltage control has also greatly hindered the application of SWCNT-FETs in the fabrication of low power consumption logic circuits^8^. Therefore, the control of hysteresis and the continuous turning of threshold voltage in the networked SWCNT-FETs are significantly important for realizing reliable SWCNT logic circuits at large scales^16^.

In this paper, the SWCNT-FETs in double-gated structure have been fabricated by using a ferroelectric poly (vinylidene fluoride-trifluoroethylene) (P(VDF-TrFE)) polymer gate insulator to obtain the controllable hysteresis of drain current under different polarized states. The schematic of our SWCNT-FETs by ferroelectric gating (SWCNT-FeFET) device is shown in Fig. 1a and the optical micrograph of these SWCNT-FeFET arrays is shown in Fig. 1b. The transistor channel is made from a random SWCNT network composed of 98% semiconducting single-walled CNTs, which is connected by a source and drain electrodes with a 10 nm thick Cr layer and 50 nm thick Au layer. The P(VDF-TrFE) film is chosen as the top gate insulator due to its high dielectric constants^17^, isolation effect of moisture^18^ and polarization properties^19^,^20^,^21^,^22^, which can compensate the current hysteresis arising from SWCNT networks and achieve a controllable hysteresis loop and threshold voltage of SWCNT channel under different polarized states. Meanwhile, the threshold voltage can be also tuned to desired values quantitatively by applying bottom gate voltage to control the carrier concentration in SWCNT network channel under the electrostatic doping effect. Meanwhile, the P(VDF-TrFE) as an ogranic polymer is soluble in ketones, which is usually used to remove photoresist in standard lithography process. Herein, we adopted the standard lithography process to achieve the graphics of organic polymers by dry etching process, which offers a great potential to the scaling down of the size of organic electronics and the compatibility with conventional CMOS process technology.

## Results

### Characterization of SWCNT-FeFETs

**Figure 1:**
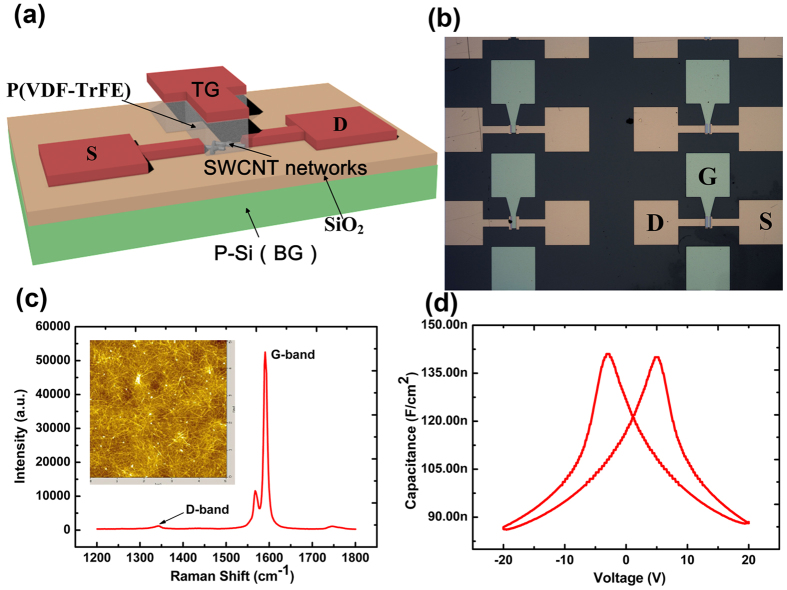
(**a**) Schematics of our fabricated double-gated SWCNT networks based FETs. (**b**) The optical image of our device arrays. (**c**) The Raman spectrum of the SWCNT networks on the Si/SiO_2_ substrate. The inset is the AFM image of SWCNT networks. (**d**) The capacitance-voltage (*C*–*V*) characteristics of Al/P(VDF-TrFE)/ITO structure.

Figure 1c shows the Raman spectrum of the SWCNT networks on the Si/SiO_2_ substrate, from which typical G and 2D bands and almost no obvious D bands can be clearly observed, indicating good quality of SWCNT networks. A densely packed SWCNT network with a density of more than 100 nanotubes per square micron on the poly-L-lysine treated SiO_2_ surface has been observed from the AFM image as shown in the inset of Fig. 1c, which is suitable for FET channel layer applications. From Fig. 1d, a symmetrical hysteresis behavior can be seen with two peak capacitance values observed at ±5 V under different voltage sweeping directions at the measurement frequency of 1.2 MHz, indicating the reversal of polarization and weakest intensity of polarization. However, the minimum capacitances have been found at ±20 V, which is induced by the saturated polarization. It demonstrates the typical hysteresis properties induced by the ferroelectric polarization of P(VDF-TrFE) films.

### Electrical properties of double-gated SWCNT-FeFETs

**Figure 2:**
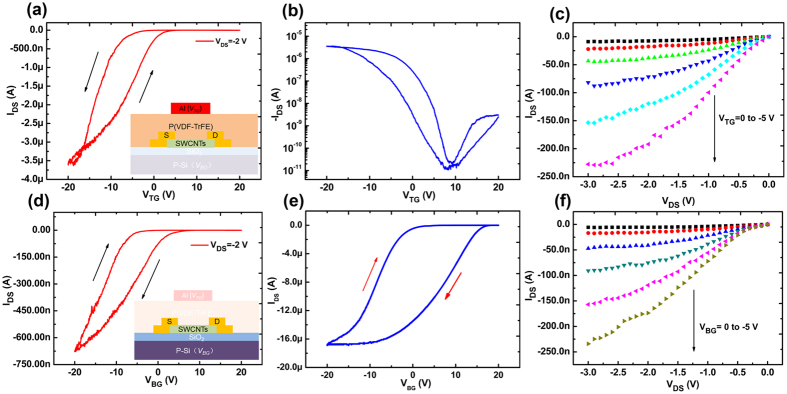
(**a**) The hysteresis characteristics of P(VDF-TrFE) top-gated SWCNT-FETs. The inset is the measured structure with top-gate electrodes applied by *V*_*TG*_. (**b**) The logarithmic curve from the (**a**). (**c**) The output characteristics of P(VDF-TrFE) top-gated SWCNT-FETs with the *V*_*TG*_ ranging from 0 to −5 V. (**d**) The transfer characteristics of SWCNT-FETs under bottom gate voltage with the top gate grounded. The inset is the measured structure with the bottom-gate electrodes applied by *V*_*BG*_. (**e**) The transfer characteristics of SWCNT-FETs under bottom gate voltage before P(VDF-TrFE) films spin-coated on the top of SWCNT network. (**f**) The output characteristics of the SWCNT-FETs with the *V*_*BG*_ ranging from 0 to −5 V.

The transfer curves represented by the drain current as a function of top-gate voltage (*I*_*DS*_*−V*_*TG*_) of the SWCNT-FeFETs under a top gate voltage sweeping from 20 to −20 V and back to 20 V as shown in Fig. 2a. The inset is the measured structure with top-gate electrodes applied by *V*_*TG*_. It indicates a typical characteristic of p-type semiconductors with SWCNT network as active layers with a forward voltage sweep from positive voltage to negative voltage. Here, the voltage difference at an average *I*_*DS*_ ((*I*_*DS, max*_* + I*_*DS, min*_)/2) has been defined as hysteresis window and an anticlockwise current hysteresis was observed with a hysteresis window of 5.6 V. The inset of Fig. 2b shows the semilog plot of the transfer curves with an on/off ratio of 4 × 10^5^ for the hole current. From Fig. 2c, it presents output curves under the modulation of the top gate effect of the device, in which *I*_*DS*_ increases with the negative *V*_*DS*_ and reaches saturation due to the pinch off of the channel, indicating that there is no obvious contact barrier in such SWCNT-FeFET device. To gain insight into the origin of the hysteresis observed in the SWCNT network channel, the *I*_*DS*_*-V*_*BG*_ transfer curves after (as shown in Fig. 2d) and before (as shown in Fig. 2e) P(VDF-TrFE) films spin-coated on the top of the SWCNT network channel have been investigated with the top electrodes grounded and back electrodes applied to a closed sweeping voltage. The inset of Fig. 2d is the measured structure with the bottom-gate electrodes applied by *V*_*BG*_. Compared the transfer characteristics by SiO_2_ gating with and without P(VDF-TrFE) layer, the hysteresis window has been obviously reduced from 16.5 V to 7.5 V and the current has been also reduced by two orders of magnitude. The decrease of clockwise hysteresis window by SiO_2_ back gating confirms the isolation effect of P(VDF-TrFE) films from the environmental water and oxygen molecules on the top surface of SWCNT networks. Meanwhile, the remaining clockwise hysteresis may be due to the charges already trapped at the surface between SiO_2_ and SWCNTs. It is noted that both the clockwise hysteresis curves by SiO_2_ back gating with and without P(VDF-TrFE) films are observed, which are obviously opposite to that by P(VDF-TrFE) top gating. The origin of the clockwise hysteresis by SiO_2_ back gating can be due to the interference of moisture or oxygen from the surrounding environment and charge trapped at the surface between SiO_2_ and SWCNT, which contributes to an unstable electrical performance. The conversion from clockwise hysteresis to anticlockwise one by P(VDF-TrFE) top gating is attributed to the ferroelectric polarization characteristics. The different polarized states controlled by the applied *V*_*TG*_ induce the change of carrier density in the SWCNT channel and the type of carrier won’t change until the reversal of polarization happens, which results in the anticlockwise hysteresis. It demonstrates that current hysteresis in a top-gate FET with a solution-processed SWCNT network is significantly suppressed and a tunable anticlockwise hysteresis has been achieved under the modulation of ferroelectric polarization. Figure 2f shows the output characteristics of the SWCNT-FETs by SiO_2_ back gating, which shows the similar p-type semiconductors properties with that by P(VDF-TrFE) top gating.

### Working mechanism of SWCNT-FeFET under the ferroelectric polarization

**Figure 3:**
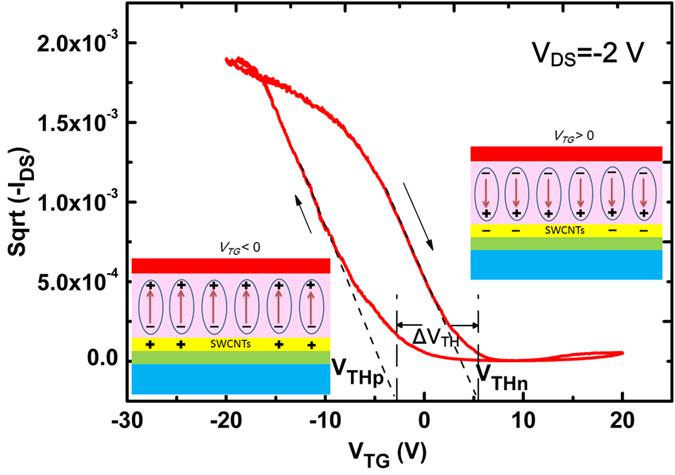
The working mechanism and the hysteresis behaviors of the P(VDF-TrFE) top gate SWCNT-FETs. The insets are the schematics of charge distribution at SWCNT/P(VDF-TrFE) interface with different polarization directions.

The SWCNT-FeFET working mechanism has been discussed as shown in the Fig. 3. In such P(VDF-TrFE) top-gated SWCNT-FETs, the ferroelectric layer has been used as the top gate dielectric layer with the SWCNT networks as the channel layer. When a negative *V*_*TG*_ is applied to the top gate, the polarization of the ferroelectric film will be aligned upward with the protons (H+) aggregating at the top surface of P(VDF-TrFE) films, whereas the electronegative fluorine anions (F−) oriented toward the surface between P(VDF-TrFE) films and the SWCNT networks. In this case, positive charges are induced into the SWCNT networks under the effective negative field effect of *V*_*TG*_. This results in the accumulation of holes in the p-type SWCNT network conducting channel to form a high conductance state, representing the ON state. The same working principle exists in the case that a positive *V*_*TG*_ is applied to the top gate and electrons are induced in the SWCNT networks as the OFF state. The hysteresis behavior of the SWCNT-FeFETs as a function of top gate voltage is shown in Fig. 3. Considering the ferroelectric hysteresis behaviors, the reversal of polarization only occurs when the applied voltage exceeds the coercive voltage of the ferroelectric layer. The threshold voltage *V*_*TH*_ of the SWCNT networks is observed as *V*_*THp*_ with the *V*_*TG*_ swept from the positive to the negative voltage and *V*_*THn*_ with the *V*_*TG*_ swept from the negative to the positive voltage. The holes induced by upward polarization and electrons induced by downward polarization in the SWCNT channel are determined by how *V*_*TG*_ will be applied on top-gate electrodes, which enables the modulation in channel conductance and threshold voltages. In this way, an effective way can be achieved to tune the threshold voltage by changing the doping concentration under different polarized states.

### Modulation effect of double-gating on the hysteresis behaviors and threshold voltage

**Figure 4:**
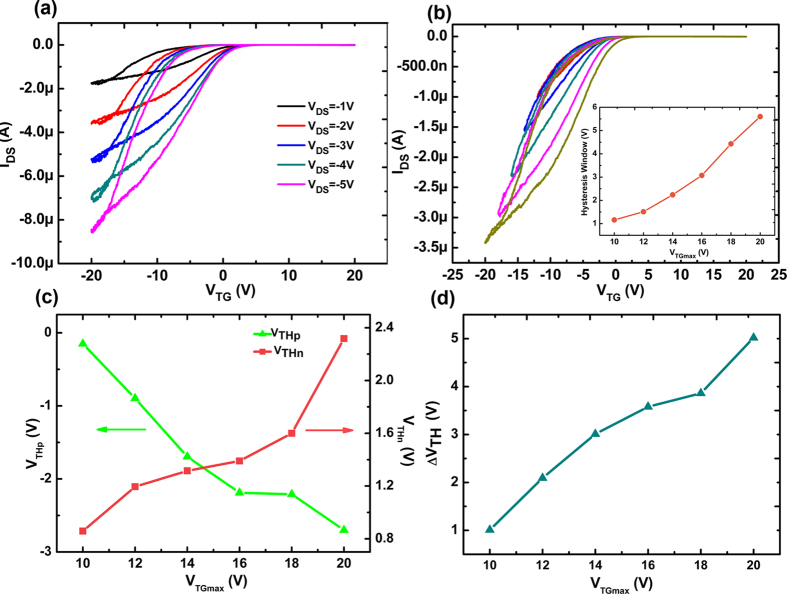
(**a**) The transfer curves of P(VDF-TrFE) top gate transistors with the *V*_*DS*_ ranging from −1 to −5 V. (**b**) The transfer curves of P(VDF-TrFE) top gate transistors with the *V*_*TG*_ sweeping ranges from −10 to −20 V. The inset shows the hysteresis window plotted as a function of *V*_*TGmax*_. (**c**) The threshold voltage (*V*_*THp*_ and *V*_*THn*_) under different sweeping directions with the *V*_*TG*_ sweeping ranges from −10 to −20 V. (**d**) The shift of *V*_*THp*_ and *V*_*THn*_ is plotted as a function of *V*_*TGmax*_.

The modulation effect of the applied voltage on the hysteresis behaviors of the SWCNT-FeFET is shown in the Fig. 4. It is noted that the clear hysteresis behaviors are observed only in the p-channel region indicating the unipolar properties of the fabricated SWCNT networks and the increase of the hysteresis with the *V*_*DS*_ ranging from −1 to −5 V, demonstrating the increasing accumulated hole carrier density at the same gate voltage as shown in Fig. 4a. The hysteresis behaviors as a function of *V*_*TG*_ under different sweeping ranges are shown in Fig. 4b. The hysteresis loop becomes enlarged when the maximum 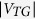 increases from 10 to 20 V at a step of 2 V. The hysteresis window becomes larger with the increase of *V*_*TG*_ sweeping range and a largest hysteresis window about 5.7 V has been achieved at *V*_*TG*_ equal to 20 V as shown in the inset of Fig. 4b, which confirms that the origin of such hysteretic behavior is due to the polarization of the ferroelectric layer^23^. In order to understand the modulation effect on the threshold voltage by the ferroelectric polarization, the *V*_*THp*_ and *V*_*THn*_ are plotted as a function of the *V*_*TGmax*_ under different sweeping ranges as shown in Fig. 4c. Increasing *V*_*TGmax*_ sweeping ranges applied on the top gate electrodes induce an enhanced arrangement of dipoles within the P(VDF-TrFE) films, which results in the increase of carrier density in SWCNT networks due to the ferroelectric polarization property of P(VDF-TrFE) films^24^. More electrons or holes will be accumulated in the SWCNT networks resulting in an n-doping or p-doping effect and the increasing shift of the threshold voltage as shown in Fig. 4d. It demonstrates the modulation effect of the ferroelectrics on the threshold voltages.

**Figure 5:**
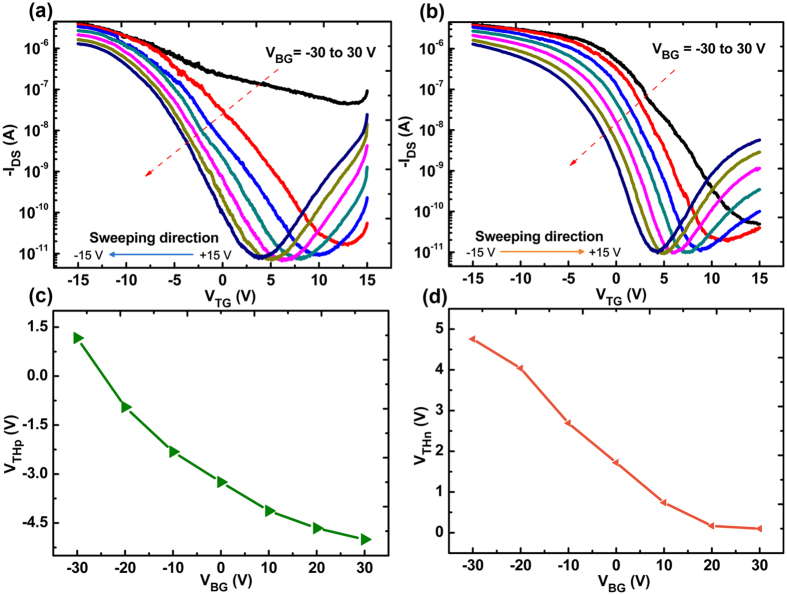
(**a**,**b**) The transfer curves of P(VDF-TrFE) top gate transistors are plotted as a function of *V*_*TG*_ with the *V*_*BG*_ ranging from −30 to 30 V under different sweeping directions. (**c**,**d**) The plot of threshold voltage (*V*_*THp*_ and *V*_*THn*_) vs *V*_*BG*_.

It is found that *V*_*TH*_ can be also continuously tuned by applying a bottom gate voltage (*V*_*BG*_) in such double-gate configuration. A clear *V*_*TH*_ shift with *V*_*BG*_ increasing from −30 to 30 V with a step of 10 V can be observed in the transfer curves of the P(VDF-TrFE) top-gated SWCNT-FETs under different *V*_*TG*_ sweeping directions (Fig. 5a,b). It can be attributed to the applied *V*_*BG*_, which changes the charge concentrations in the channel and thus modifies the shift of the threshold voltages. From Fig. 5c,d, it can be seen that when the *V*_*BG*_ changes from −30 to 30 V, the induced charges in the channel varies from holes to electrons resulting in a p-doping shifting to n-doping with the *V*_*THp*_ from 1.25 to −4.9 V and *V*_*THn*_ from 4.8 nearly to 0 V. As noted, precisely controlling the transistor threshold voltage for realizing high noise-margin and robust circuits remains to be challenging. Here, an external field effect by *V*_*BG*_ has been proposed to continuously tune the threshold voltage of SWCNT networks, which helps SWCNT network based circuits to have a high immunity towards the electrical noise in a system^7^.

## Discussion

The double-gated structural field effect transistors with SWCNT networks as active channel and solution processed P(VDF-TrFE) as top gating insulator have been achieved. The initial hysteresis behaviors of SWCNT networks caused by charge traps from the ambient environment can be effectively suppressed and a controllable hysteresis has been achieved under the polarization effect of P(VDF-TrFE). Meanwhile, controlling the threshold voltage of the SWCNT-FETs can also be achieved by modifying the polarized states of the top gate dielectric P(VDF-TrFE). Moreover, bottom gating induced electrostatic doping effects are found to be another effective way to continuously tune the threshold voltage in the SWCNT-FETs by modulating the doping level in the SWCNT channel. The tunable threshold voltage by the polarization of ferroelectric and the electrostatic doping effect of bottom gate voltage is highly beneficial for the transistor operation in robust circuits.

## Method

### Fabrication of double-gate SWCNT-FeFETs

Single crystal p-Si (100) substrate (2–3 Ω·cm) was used as the bottom gate electrodes with upper 90 nm silicon oxide (SiO_2_) bottom gate insulator by thermal growth. The S/D electrodes consisted of a 10 nm chrome (Cr) adhesion layer followed by 50 nm gold (Au) were patterned by standard photolithography procedures and deposited by electron beam evaporation. Then Poly-L-lysine (0.1% w/v in H_2_O, Sigma-Aldrich) solution was drop-cast onto SiO_2_ surface for 5 min to functionalize the SiO_2_ surface for SWCNT deposition, followed by immersing the substrate in 98% semiconductor-enriched SWCNT suspension (NanoIntegris Inc.) for 1 hour and a rinse with DI water and isopropanol to remove the excess solution. Then standard photolithography and the following oxygen plasma etching were used to define the channel region by removing the extra SWCNTs. At this time, a bottom-gated SWCNT-FET by SiO_2_ gating has been fabricated. Then, the precursor solution by dissolving the P(VDF-TrFE) (70/30) pellets in Diethyl carbonate was spin-coated on the top of the SWCNT-FETs fabricated above. To improve the degree of crystallization, the as-prepared P(VDF-TrFE) films with 200 nm thickness were annealed at 135 °C for 30 min. In order to define the top gate electrodes, 50 nm thick Al were thermally evaporated on the top of P(VDF-TrFE) films before photolithographic patterning. Then, another standard photolithography was used to define the top gate electrode and aluminum etching solution was used to remove the Al without the protection of photoresist. Finally, oxygen plasma etching was used to remove the remnant photoresist as well as the organic ferroelectric films outside the top gate electrodes protection.

### Characterization

In order to examine the surface morphologies of the as-prepared SWCNT network films, the microstructure and morphology of the SWCNT network films were observed by atomic force microscopy (AFM, SPA 500, Seiko Instruments Inc.) with taping mode. The capacitance-voltage (*C*–*V*) characteristics of Al/P(VDF-TrFE)/ITO structure has been investigated to exam the hysteresis properties of the P(VDF-TrFE) films. The electrical properties of the SWCNT-FeFETs by ferroelectric gating were measured by Aligent 1500 semiconductor characterization system. The current-voltage (*I*–*V*) characteristics of the SWCNT-FeFETs were measured with the top electrodes applied to a sweeping voltage in a closed hoop and the bottom electrode grounded.

## Additional Information

**How to cite this article**: Sun, Y.-L. *et al*. Controllable Hysteresis and Threshold Voltage of Single-Walled Carbon Nano-tube Transistors with Ferroelectric Polymer Top-Gate Insulators. *Sci. Rep*. **6**, 23090; doi: 10.1038/srep23090 (2016).
